# The performance of approximations of farm contiguity compared to contiguity defined using detailed geographical information in two sample areas in Scotland: implications for foot-and-mouth disease modelling

**DOI:** 10.1186/1746-6148-9-198

**Published:** 2013-10-08

**Authors:** Jessica S Flood, Thibaud Porphyre, Michael J Tildesley, Mark EJ Woolhouse

**Affiliations:** 1 Epidemiology Group, Centre for Immunity, Infection and Evolution, School of Biological Sciences, University of Edinburgh, Kings Buildings, West Mains Road, Edinburgh EH9 3JT, UK; 2 Centre for Complexity Science, Mathematics Institute, Zeeman Building, University of Warwick, Coventry CV4 7AL, UK

## Abstract

**Background:**

When modelling infectious diseases, accurately capturing the pattern of dissemination through space is key to providing optimal recommendations for control. Mathematical models of disease spread in livestock, such as for foot-and-mouth disease (FMD), have done this by incorporating a transmission kernel which describes the decay in transmission rate with increasing Euclidean distance from an infected premises (IP). However, this assumes a homogenous landscape, and is based on the distance between point locations of farms. Indeed, underlying the spatial pattern of spread are the contact networks involved in transmission. Accordingly, area-weighted tessellation around farm point locations has been used to approximate field-contiguity and simulate the effect of contiguous premises (CP) culling for FMD. Here, geographic data were used to determine contiguity based on distance between premises’ fields and presence of landscape features for two sample areas in Scotland. Sensitivity, positive predictive value, and the True Skill Statistic (TSS) were calculated to determine how point distance measures and area-weighted tessellation compared to the ‘gold standard’ of the map-based measures in identifying CPs. In addition, the mean degree and density of the different contact networks were calculated.

**Results:**

Utilising point distances <1 km and <5 km as a measure for contiguity resulted in poor discrimination between map-based CPs/non-CPs (TSS 0.279-0.344 and 0.385-0.400, respectively). Point distance <1 km missed a high proportion of map-based CPs; <5 km point distance picked up a high proportion of map-based non-CPs as CPs. Area-weighted tessellation performed best, with reasonable discrimination between map-based CPs/non-CPs (TSS 0.617-0.737) and comparable mean degree and density. Landscape features altered network properties considerably when taken into account.

**Conclusion:**

The farming landscape is not homogeneous. Basing contiguity on geographic locations of field boundaries and including landscape features known to affect transmission into FMD models are likely to improve individual farm-level accuracy of spatial predictions in the event of future outbreaks. If a substantial proportion of FMD transmission events are by contiguous spread, and CPs should be assigned an elevated relative transmission rate, the shape of the kernel could be significantly altered since ability to discriminate between map-based CPs and non-CPs is different over different Euclidean distances.

## Background

Despite implementation of a national livestock movement ban 3 days after the first confirmed case of foot-and-mouth (FMD) in the UK in 2001, the disease continued to spread through the farming landscape [1]. Such spread is thought to have occurred mainly by nose-nose contact of livestock across shared fence lines and by contaminated fomites carried on people, vehicles, machinery, or blown by wind between premises [1]. Mathematical models were developed in order to capture the likely spread through space, to predict the likely impact of control strategies, and consequently to inform disease control policies implemented [2,3]. To describe the spatial pattern of spread, a transmission kernel was incorporated into the model. This kernel described the decay in rate of transmission to susceptible livestock premises with increasing Euclidean distance from an infected premises (IP) source (calculated between farm premises point locations). For the model of Keeling *et al*. this was derived from infection tracing following the livestock movement ban [3]. While this model captured the regional pattern of spread well, accuracy at the individual farm level was low for IPs, with about 12% of reported case premises over the duration of the epidemic being captured by simulations [4]. Although this low accuracy is in part due to stochastic variation, assumed homogeneity of the landscape by the kernel is also likely to have contributed.

In addition to incorporating space by using the spatial transmission kernel, contiguous premises (CPs) (farm premises neighbouring infected premises which were at highly elevated risk of infection) were modelled by area-weighted tessellation in order to examine the likely effect of culling CPs [3,5]. Area-weighted tessellation uses the known land areas and the known point locations of premises to construct weighted Voronoi polygons around the points. Voronoi polygons are constructed by connecting the perpendicular bisectors of lines between pairs of points, where only the closest bisectors are considered. This results in tessellated polygons, where any point within a polygon will be closer to the point around which the polygon was constructed than any other. Area-weighting this process means that the square-root of the known land area of each point pulls or pushes the perpendicular bisector towards or away from a point, depending on the comparative size of the square-root of the paired farm’s area. Contiguity is then based on having a shared polygon edge. This technique was applied to Great Britain’s farm premises, as recorded by the June 2000 agricultural census, to determine which farms were contiguous to other farms, and culling of CPs within model simulations were determined on this basis [3].

Based in part on the outputs of these models, pre-emptive culling of livestock contiguous to infected premises (IPs), livestock thought to be dangerous contacts (DCs) of an IP, and livestock within 3 km of/local to an IP was performed in 2001 [5]. While this control strategy did eventually bring the epidemic to a halt, it has been suggested that it could have been better targeted to reduce the epidemic duration and impact since it appeared that, as implemented in practice, low risk premises were targeted over higher risk premises [6]. Additionally, heterogeneities in the fragmentation of the livestock farming landscape across the country suggest that some regions did not require pre-emptive culls for disease containment [7]. The epidemic cost the UK economy approximately £6bn [8]. Thus, appropriate control strategies are necessary to reduce any future epidemic’s impact in terms of the number of livestock affected and the cost to the economy. Greater predictive accuracy of mathematical models may increase trust, and consequently compliance with suggested control strategies in practice. The 2001 FMD transmission kernel developed by Keeling and collaborators indicated that approximately 50% of transmission occurred within 3 km of an IP after the implementation of a livestock movement ban [9] – thus local spread is important, but there is a lack of understanding as to how this is related to true contiguity.

While the approximations used in the models will clearly, to some degree, capture the essence of spatial proximity, they are yet to be assessed for their accuracy in this respect. A kernel based on Euclidean distance between point locations not only fails to recognise that farms in reality are areas, but also that the landscape is non-homogenous and that transmission potential is therefore not equal in all directions. Although area-weighted Voronoi polygons consider farms as areas, these are derived from point locations and therefore may not reflect how farms share boundaries in reality. Additionally, geographical features such as rivers, ditches and railways may act as barriers to transmission, and therefore prevent contiguity in terms of disease transmission [10].

We consider that the level of risk a premises is perceived to be at, based on its point distance from an IP, may be altered by knowing actual premises contiguity, particularly in the case of contact spread diseases such as FMD since the distance between two farm point locations may be considerable despite their fields actually being in contact. Thus, at the extreme end of the spectrum, the decay in risk with increasing Euclidean distance may simply explain the distribution of point distances between actual CPs.

Different methods of incorporating the spatial arrangement of farm premises into mathematical models of infectious diseases among livestock may have considerable impact on predicted epidemic size, distribution, and optimal control strategies. Therefore, this paper aims to compare the properties of the contact networks that arise from the classification of farm premises as being in contact by point distance measures, by Voronoi and area-weighted Voronoi tessellation, and by maps showing the field boundaries of premises and geographical features that surround them. Additionally, how well approximation methods capture farm premises considered to be in contact (the term CP will be used to describe contact) according to field edge distance and presence of geographical features will be assessed. Another measure based solely on distance between the closest field edges of premises will also be added to the comparison as such measures have recently been used in statistical analysis of bovine tuberculosis persistence [11]. Areas in Ayrshire and Aberdeenshire were chosen to evaluate these measures since they are both important livestock farming areas, but with different farm types dominating: Ayrshire consisting mainly of dairy cattle farming, and Aberdeenshire consisting of a mixture of cattle (mainly beef), sheep, pig and crop production [12,13].

## Methods

Spatial data were visualised and manipulated in ArcGIS version 9.3 (ESRI, Redlands, CA, USA). Farm premises point locations were obtained from the Animal Health and Veterinary Laboratories Agency (AHVLA). Fields of farm premises were obtained from the Integrated Administration and Control System (IACS) dataset from 2006. The June 2006 Agricultural Census data was matched to the point location data based on the county-parish-holding (CPH) number to select only premises with any cattle, sheep or pigs. A sample study area was then selected within each of Aberdeenshire and Ayrshire based on the point locations of premises being within an area of approximately 15x15 km. The point locations of these premises were then matched up with the IACS field data based on the parish-holding (PH) component of the CPH number. The distance between PH-matched point and field locations were calculated using the ArcGIS ‘Generate Near Table’ tool.

Ordnance Survey (OS) MasterMap® Topography Layer data, at a varying scale of 1:1250 to 1:10000, was used to map geographical features. The OS MasterMap® data used for Ayrshire was provided direct from the OS (updated on 23/08/2012), whereas for Aberdeenshire the data was downloaded from EDINA Digimap (EDINA Digimap Ordnance Survey Service <http://edina.ac.uk/digimap>, downloaded March 2012, updated on 08/06/2011). For Ayrshire roads were indicated by topographic lines where DescGroup = “Road Or Track”, and tracks by topographic areas where Theme = “Roads Tracks And Paths”; for Aberdeenshire roads and tracks were indicated by topographic lines where Theme = “Land; Roads Tracks And Paths”. In both sample areas rivers >2 m wide were indicated by sets of double topographic lines where DescGroup = “Inland Water”, and inland water courses ≤2 m wide (henceforth referred to as small rivers/ditches) were indicated by single topographic lines where DescGroup = “Inland Water”. Railways were indicated by topographic lines where Theme = “Rail”.

### Defining Contiguous Premises (CPs)

For each of the Aberdeenshire and Ayrshire samples a dataset was then created whereby every premises was paired to every other premises within 7 km of it, in terms of Euclidean distance between point locations. From this dataset each premises pair was then classified as being contiguous or not contiguous according to eight CP approximation definitions:

a) <1 km distance between point locations of premises;

b) <3 km distance between point locations of premises;

c) <5 km distance between point locations of premises;

d) <26 m distance between premises field edges at their closest point;

e) <151 m distance between premises field edges at their closest point;

f) <1 km distance between premises field edges at their closest point;

g) sharing a Voronoi polygon edge;

h) sharing an area-weighted Voronoi polygon edge.

The Voronoi polygons were generated from the point locations in ArcGIS. A wider sample of points was used to create the Voronoi polygons to act as a buffer so that within-sample the polygons were not influenced by edge effects. This dataset was checked for occurrences where point locations were shared by different premises. These could arise where two premises shared the same postcode, and where each premises’ point location was derived from that postcode. Where this happened, the pairs were taken to be CPs with each other, and to have identical other CPs. The area-weighted Voronoi polygons were weighted by known premises area. This was scripted and run in MATLAB (The MathWorks, Inc., Nat- ick, MA, USA). Distances between point locations, field boundaries, and shared Voronoi polygon edges were calculated using the ArcGIS ‘Generate Near Table’ tool.

Maps of IACS and OS MasterMap data were checked visually to assess whether each premises pair actually shared a fence boundary, had fence boundaries separated by <15 m, were separated by a road/track or railway, were divided by a river or by a small river/ditch. The entire length of each premises boundary was considered. The relative length of each type of separation between premises was not considered such that if the premises shared a boundary at any point, they were classified as having a shared boundary, regardless of the boundary length. For classification in terms of separation by landscape features, the premises pairs would only be classified as such if the entire length of the shared boundary appeared to be separated by this feature. In cases where premises were separated along the entire boundary by more than one types of geographic feature, but where each feature type did not run the entire length of the boundary, the feature with the lowest perceived ‘barrier effect’ was taken to be the feature of separation (small river/ditch < road/track < river). Only one premises pair had a railway line running the entire length of their shared boundary in Ayrshire, and no premises were separated by railway in Aberdeenshire. Thus separation by railways was not included for the purposes of this analysis.

Based on map inspection, nine further definitions of being contiguous were then considered:

(i) having any fields separated up to a maximum distance of 15 m;

(ii) having any fields separated up to a maximum distance of 15 m not including premises divided by a river;

(iii) having any fields separated up to a maximum distance of 15 m not including premises separated by a road/track;

(iv) having any fields separated up to a maximum distance of 15 m not including premises divided by a river or separated by a road/track;

(v) having any fields separated up to a maximum distance of 15 m not including premises divided by a river or small river/ditch or separated by a road/track;

(vi) having any fields separated up to a maximum distance of 15 m not including premises divided by a river or small river/ditch;

(vii) having fields with a shared boundary (i.e. no separation);

(viii) having fields with a shared boundary not including premises divided by a river;

(ix) having fields with a shared boundary not including premises divided by a river or small river/ditch.

The cumulative number of map-based CPs, according to the nine definitions (i-ix) listed above, with 0.25 km increases in Euclidean point distance was calculated.

### Measuring agreement between the different CP definitions

Symmetric matrices of the premises in the samples were produced for each of the seventeen definitions of contiguity (approximation methods a-h, and map-based methods i-ix) using R version 2.13.2 (R Development Core Team, Vienna, Austria, <http://www.R-project.org/>). Each element took the value 0 or 1 depending on whether the premises pairs were non-contiguous or contiguous under the definition, respectively. Agreement between matrices of different CP definitions was estimated using four measures: concordance, sensitivity (Se), positive predictive value (PPV), and True Skill Statistic (TSS), where:

● Concordance = (TP + TN)/ (TP + FP + FN + TN),

● Se = TP / (TP + FN),

● PPV = TP / (TP + FP),

● TSS = (sensitivity + specificity - 1); where Specificity = TN / (FP + TN), and where TP = true positive, FP = false positive, TN = true negative, FN = false negative.

Concordance, Se and PPV were multiplied by 100 to give a percentage.

Calculating Se of point distance, field edge distance, and tessellation measures against a ‘gold standard’ of map-based contiguity as defined by field edge separation and landscape features, enabled us to study how many farm premises were missed by the approximation methods that were contiguous under the map-based definitions (by identifying the proportion of map-based CPs that were correctly identified by each method). PPV enabled us to examine how many farm premises the approximation methods picked up that were not actually contiguous, by giving the proportion of approximation method CPs that were contiguous under the map-based definitions. TSS gave an overall assessment of how well the approximation methods discriminated between contiguous and non-contiguous premises pairs as defined by map-based methods.

TSS was used in preference to Kappa as it provides a similar measure of accuracy of the discrimination of two methods for a binary outcome, without being affected by prevalence [14]. This measure, also known as the Hanssen and Kuipers statistic and Youden’s Index, has values ranging from −1 to +1 and has previously been used to assess the accuracy of weather prediction models [15-18].

The methodology used means that there was some room for human error in the classification of contiguity based on presence of landscape features along or between farm premises boundaries. To minimise this, the boundaries of CP pairs were checked twice, and the symmetry of the resulting matrices was verified using the command ‘isSymmetric’ in R, with maps being re-checked in the event of apparent asymmetry.

### Network properties of different CP definitions

Network density and mean degree were calculated for a subset of the contiguous definitions. Density was calculated using the ‘igraph’ package in R, and was calculated on the sample premises only. In order to correct for edge effects in the calculation of mean degree, new data sets were created to count all CPs associated with farm premises within the sample, rather than only other premises from within the sample. For field edge based contiguity, all premises with fields listed in IACS with any cattle, sheep or pigs were included (this meant there were some premises within the sample zone not previously included as they did not belong to a point location within the selected area). For point distance based contiguity, all premises with any cattle, sheep or pigs and point locations that matched up to IACS field data were included. Mean degree was calculated by species kept on holding for the categories that had ≥5 holdings in, for all map-based CP definitions and area-weighted tessellation.

## Results

In the Aberdeenshire sample 113 premises points were first selected, but only 107 (94.7%) could be linked to fields within the IACS database. Of these point locations, 98 (91.6%) were sourced from an address match, 6 (5.6%) from a postcode match and 3 (2.8%) from the parish centroid. Four pairs of premises shared identical point locations; three of these were sourced from address matches, and one from a postcode match. For the Ayrshire sample 197 premises points were first selected, of which only 184 (93.4%) could be linked to fields within the IACS database. Of these point locations, 156 (84.8%) were sourced from an address match, 20 (10.9%) from a postcode match and 8 (4.3%) from the parish centroid. Seven pairs and one triplet of premises shared identical point locations. Five of the pairs with identical point locations were sourced from an address match, and one from a postcode match.

In the Aberdeenshire sample, 88.8% (n = 95) of premises point locations were <60 m from their CPH-matched nearest field; 2.8% (n = 3) were separated by 60-1000 m, and the remaining 8.4% (n = 9) by ≥1000 m. In the Ayrshire sample, 83.7% (n = 154) had point locations <60 m from their CPH-matched nearest field, while 8.2% (n = 15) were separated by 60-1000 m, and 8.2% (n = 15) by ≥1000 m. The least accurate of the point location sources was the parish centroid, followed by the postcode. The distribution of the PH-matched point-field distances by the point location information source can be seen in Additional file 1.

The majority of premises in the Ayrshire sample kept cattle only (70.1%), and no premises kept any pigs (Table 1). The median area of the farm premises was 73.5 hectares (IQR: 51.9-104.8), with a median of 16 fields (IQR: 11–22) (mean = 17.7). In the Aberdeenshire sample 47.7% of all premises kept cattle and sheep, while just over a third kept cattle only (34.6%), and only six holdings kept pigs (Table 1). The median area of the farm premises was 76.4 hectares (IQR: 40.0-174.0), with a median of 19 fields (IQR: 11–32) (mean = 22.0).

**Table 1:** Distribution of types of livestock kept on premises in samples

**Animals kept on holding**	**Aberdeenshire**		**Ayrshire**	
	**Number**	**%**	**Number**	**%**
**Cattle only**	37	34.6	129	70.1
**Sheep only**	13	12.1	16	8.7
**Pigs only**	1	0.9	0	0.0
**Cattle/sheep**	51	47.7	39	21.2
**Cattle/pigs**	1	0.9	0	0.0
**Sheep/pigs**	1	0.9	0	0.0
**Cattle/sheep/pigs**	3	2.8	0	0.0
**Total**	107	100.0	184	100.0

### Agreement between the different CP definitions

**Figure 1:**
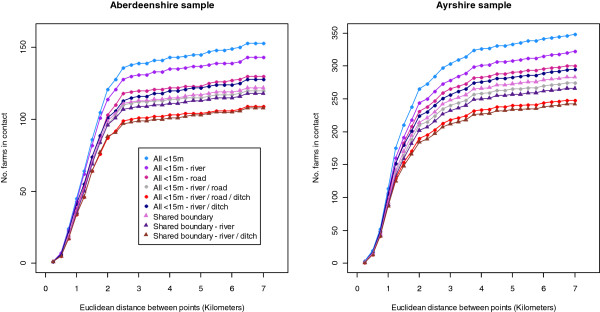
Number of premises in contact by map-based measures up to 7 km point distance.

Considering farms to be contiguous if they lie within 7 km Euclidean distance of one another’s point locations captured 98.1% (153/156) and 97.8% (348/356) of CP premises pairs that were separated by <15 m at their field edges in Aberdeenshire and Ayrshire, respectively. The pattern of map-based CP identification over increasing Euclidean distance between the premises point locations differed slightly between Aberdeenshire and Ayrshire (Figure 1). In Aberdeenshire, the number of map-based CPs identified began to plateau at 2.5 km point distance, such that 88.9% (n = 136) of premises separated by <15 m at their field edges were captured within 2.5 km. In Ayrshire however, the plateau was less distinct, and began at around 3.25 km; 88.8% (n = 309) of premises separated by <15 m at their field edges were captured by this distance.

Concordance of approximation measures was very high for point distances <1 km, field edge distances <1 km, and Voronoi and area-weighted tessellation for both Aberdeenshire and Ayrshire (all >87% agreement with map-based contiguity measures) (Additional file 2). This however was distinctly biased towards non-contiguous pair agreements (True Negatives).

Sensitivity was therefore calculated to find the proportion of map-based CPs that were correctly identified by the approximation methods. Sensitivity was fairly consistent between map-based contiguity measures. For measures based on point distances, sensitivity was low for <1 km, only reaching >94% at point distances <5 km (Table 2). Ayrshire had a higher average sensitivity at <1 km point distance compared to Aberdeenshire (Ayrshire 33.8%; Aberdeenshire 30.3%), but lower average sensitivity at <3 km point distance (Ayrshire 87.4%; Aberdeenshire 92.0%). Both samples reached an average of about 96% sensitivity at 5 km point distance. The two tessellation methods identified a higher average of map-based CPs in Aberdeenshire (Voronoi tessellation = 73.6%; area-weighted tessellation = 83.4%) than in Ayrshire (Voronoi tessellation = 63.5%; area-weighted tessellation = 68.0%). Field edge distance measures were 100% sensitive by definition (Table 2).

**Table 2:** Sensitivity (%) of approximation methods versus map-based measures for sample areas in Aberdeenshire and Ayrshire

		**Current method**							
		**All premises <1 km point distance**	**All premises <3 km point distance**	**All premises <5 km point distance**	**All premises <26 m field distance**	**All premises <151 m field distance**	**All premises <1 km field distance**	**By Voronoi tessellation**	**By area weighted tessellation**
**Aberdeenshire**									
**Gold standard**	**All <15 m**	29.4	90.8	94.8	100.0	100.0	100.0	73.2	82.4
	**All <15 m - river**	30.1	91.6	95.8	100.0	100.0	100.0	73.4	82.5
	**All <15 m - road**	29.2	92.3	94.6	100.0	100.0	100.0	73.1	83.1
	**All <15 m - river/road**	30.0	93.3	95.8	100.0	100.0	100.0	73.3	83.3
	**All <15 m - river/road/ditch**	31.2	92.7	95.4	100.0	100.0	100.0	72.5	83.5
	**All <15 m - river/ditch**	32.0	90.6	95.3	100.0	100.0	100.0	72.7	82.8
	**Shared fence**	29.5	92.6	95.9	100.0	100.0	100.0	75.4	84.4
	**Shared fence - river**	29.7	92.4	95.8	100.0	100.0	100.0	74.6	83.9
	**Shared fence - river/ditch**	31.5	91.7	95.4	100.0	100.0	100.0	74.1	84.3
**Ayrshire**									
**Gold standard**	**All <15 m**	32.8	87.1	95.7	100.0	100.0	100.0	62.6	67.2
	**All <15 m - river**	33.2	86.3	95.7	100.0	100.0	100.0	62.4	67.1
	**All <15 m - road**	32.3	88.3	96.7	100.0	100.0	100.0	64.3	69.0
	**All <15 m - river/road**	32.8	87.6	96.7	100.0	100.0	100.0	64.2	69.0
	**All <15 m - river/road/ditch**	35.5	87.9	96.8	100.0	100.0	100.0	64.9	69.0
	**All <15 m - river/ditch**	35.6	86.8	95.9	100.0	100.0	100.0	63.1	67.1
	**Shared fence**	32.9	87.6	96.5	100.0	100.0	100.0	62.5	67.5
	**Shared fence - river**	33.5	87.2	96.6	100.0	100.0	100.0	63.2	68.0
	**Shared fence - river/ditch**	36.0	87.6	96.7	100.0	100.0	100.0	64.0	68.2

PPV identified the proportion of approximation method CPs that were CPs under map-based methods, so that a low value indicates that only a low proportion of those identified are map-based CPs. For both samples PPV was consistently low (<50%) through the different map-based CP definitions for point distances <3 km and <5 km, field edge distance <1 km, and Voronoi and area-weighted tessellation (Table 3). For point distances <1 km, Aberdeen had a higher average PPV of 55.1% compared to Ayrshire which had an average PPV of 48.1%. As expected, the highest PPV was for field edge distance <26 m, and this was similar between the two samples (Aberdeenshire range 66.3-93.9%; Ayrshire range 66.9-96.1%).

**Table 3:** PPV (%) of approximation methods versus map-based measures for sample areas in Aberdeenshire and Ayrshire

		**Current method**							
		**All premises <1 km point distance**	**All premises <3 km point distance**	**All premises <5 km point distance**	**All premises <26 m field distance**	**All premises <151 m field distance**	**All premises <1 km field distance**	**By Voronoi tessellation**	**By area weighted tessellation**
**Aberdeenshire**									
**Gold standard**	**All <15 m**	65.2	22.7	10.4	93.9	85.0	37.4	38.1	36.4
	**All <15 m - river**	62.3	21.4	9.9	87.7	79.4	35.0	35.7	34.1
	**All <15 m - road**	55.1	19.6	8.9	79.8	72.2	31.8	32.3	31.2
	**All <15 m - river/road**	52.2	18.3	8.3	73.6	66.7	29.3	29.9	28.9
	**All <15 m - river/road/ditch**	49.3	16.5	7.5	66.9	60.6	26.7	26.9	26.3
	**All <15 m - river/ditch**	59.4	18.9	8.8	78.5	71.1	31.3	31.6	30.6
	**Shared fence**	52.2	18.4	8.4	74.8	67.8	29.8	31.3	29.8
	**Shared fence - river**	50.7	17.8	8.1	72.4	65.6	28.9	29.9	28.6
	**Shared fence - river/ditch**	49.3	16.2	7.4	66.3	60.0	26.4	27.2	26.3
**Ayrshire**									
**Gold standard**	**All <15 m**	56.7	17.2	7.4	96.1	80.4	32.0	41.2	39.1
	**All <15 m - river**	53.2	15.8	6.8	89.0	74.4	29.6	38.0	36.1
	**All <15 m - road**	48.3	15.0	6.4	82.9	69.3	27.5	36.5	34.6
	**All <15 m - river/road**	44.8	13.6	5.9	75.7	63.3	25.2	33.3	31.6
	**All <15 m - river/road/ditch**	43.8	12.4	5.3	68.5	57.3	22.8	30.4	28.5
	**All <15 m - river/ditch**	52.2	14.5	6.3	81.5	68.1	27.1	35.2	33.1
	**Shared fence**	46.3	14.1	6.0	78.2	65.4	26.0	33.5	31.9
	**Shared fence - river**	44.3	13.1	5.7	73.5	61.4	24.4	31.8	30.2
	**Shared fence - river/ditch**	43.3	12.0	5.2	66.9	55.9	22.2	29.3	27.5

The highest TSS scores were found for the field edge distance measures (Table 4). Out of point distance measures, <3 km had the highest TSS score (Aberdeenshire range 0.686-0.712; Ayrshire range 0.662-0.680). Point distances of <5 km and <1 km had average TSS scores of 0.393 and 0.289 in Aberdeenshire and 0.390 and 0.324 in Ayrshire, respectively. Voronoi and area-weighted tessellation had average TSS scores of 0.647 and 0.727 in Aberdeenshire and 0.588 and 0.626 in Ayrshire, respectively.

**Table 4:** TSS of different definitions of being contiguous for sample areas in Aberdeenshire and Ayrshire

		**Current method**							
		**All premises <1 km point distance**	**All premises <3 km point distance**	**All premises <5 km point distance**	**All premises <26 m field distance**	**All premises <151 m field distance**	**All premises <1 km field distance**	**By Voronoi tessellation**	**By area weighted tessellation**
**Aberdeenshire**									
**Gold standard**	**All <15 m**	0.283	0.696	0.391	0.996	0.988	0.885	0.651	0.725
	**All <15 m - river**	0.289	0.701	0.400	0.991	0.984	0.881	0.650	0.724
	**All <15 m - road**	0.279	0.705	0.385	0.985	0.978	0.876	0.643	0.725
	**All <15 m - river/road**	0.285	0.712	0.397	0.981	0.974	0.872	0.642	0.725
	**All <15 m - river/road/ditch**	0.297	0.702	0.390	0.976	0.969	0.868	0.630	0.723
	**All <15 m - river/ditch**	0.308	0.686	0.392	0.984	0.977	0.876	0.638	0.722
	**Shared fence**	0.281	0.705	0.398	0.982	0.974	0.873	0.665	0.737
	**Shared fence - river**	0.282	0.702	0.395	0.980	0.973	0.872	0.655	0.730
	**Shared fence - river/ditch**	0.299	0.691	0.390	0.976	0.968	0.868	0.647	0.731
**Ayrshire**									
**Gold standard**	**All <15 m**	0.316	0.672	0.386	0.998	0.988	0.899	0.584	0.623
	**All <15 m - river**	0.320	0.662	0.385	0.995	0.985	0.896	0.580	0.619
	**All <15 m - road**	0.309	0.680	0.394	0.992	0.982	0.893	0.598	0.637
	**All <15 m - river/road**	0.314	0.670	0.393	0.988	0.979	0.890	0.595	0.635
	**All <15 m - river/road/ditch**	0.340	0.671	0.392	0.985	0.975	0.887	0.600	0.632
	**All <15 m - river/ditch**	0.343	0.664	0.386	0.991	0.981	0.893	0.584	0.617
	**Shared fence**	0.314	0.672	0.391	0.989	0.980	0.891	0.578	0.620
	**Shared fence - river**	0.320	0.666	0.392	0.987	0.978	0.889	0.583	0.624
	**Shared fence - river/ditch**	0.344	0.668	0.391	0.984	0.974	0.886	0.590	0.624

### Network properties

**Figure 2:**
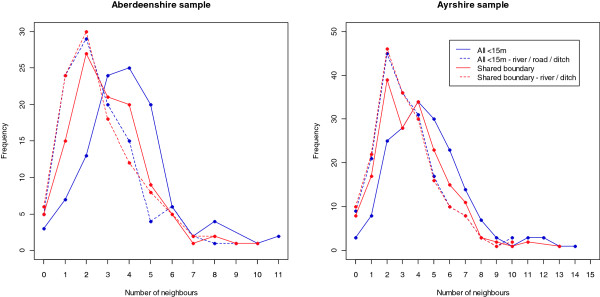
Frequency distributions of number of neighbours according to different definitions of map-based contiguity.

**Figure 3:**
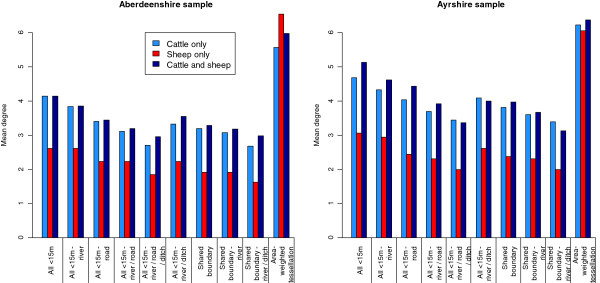
Mean degree by species kept on holding, under different definitions of contiguity.

The mean degree (i.e. mean number of CPs) was slightly higher in Ayrshire than in Aberdeenshire for all definitions of contact (Table 5). Overall, the mean degree range for the Aberdeenshire sample was 2.67-3.92 and for the Ayrshire sample was 3.21-4.64, for all map-based CP definitions. The mean degree of CPs defined as those <15 m separated at their field boundaries dropped by 1.22 and 1.34 in Aberdeenshire and Ayrshire, respectively, when the presence of all landscape features (rivers, ditches and roads/tracks) were taken to restrict contact (distribution shown in Figure 2). For CPs defined by having a shared boundary, the presence of rivers and ditches reduced the mean degree by 0.40 and 0.51 in Aberdeenshire and Ayrshire, respectively (distribution shown in Figure 2). For the point distance CP definitions, <1 km considerably underestimated mean degree when compared to map-based CP definitions, particularly in Aberdeenshire, whereas <3 km considerably overestimated it, particularly in Ayrshire. Area-weighted tessellation also overestimated mean degree compared to map-based CP definitions, although to a lesser extent than <3 km point distance. Holdings that kept only sheep had a mean degree between 0.85-1.52 and 1.13-2.07 less than holdings that kept cattle only or cattle and sheep, in Aberdeenshire and Ayrshire respectively, across all map-based CP definitions. Area-weighted tessellation (Figure 3) and point distance measures (not shown) did not identify this difference.

**Table 5:** Network properties according to different contiguity definitions for farm premises in Aberdeenshire and Ayrshire

**Contiguous classification**	**Aberdeenshire sample**		**Ayrshire sample**	
	**Mean degree**	**Density**	**Mean degree**	**Density**
**All <15 m**	3.92	0.027	4.64	0.021
**All <15 m - river**	3.64	0.025	4.27	0.019
**All <15 m - road**	3.27	0.023	3.98	0.018
**All <15 m - river/road**	3.01	0.021	3.62	0.016
**All <15 m - river/road/ditch**	2.70	0.019	3.30	0.015
**All <15 m - river/ditch**	3.26	0.023	3.94	0.018
**Shared field edge**	3.07	0.022	3.72	0.017
**Shared fence - river**	2.95	0.021	3.51	0.016
**Shared fence - river/ditch**	2.67	0.019	3.21	0.014
**<1 km distance between point locations**	1.36	0.012	2.26	0.012
**<3 km distance between point locations**	13.61	0.108	21.49	0.105
**Area-weighted tessellation**	5.95	0.061	6.25	0.036

Aberdeenshire had a higher density than Ayrshire for each definition except <1 km point distance, for which the two samples were equal (Table 5). The range of density values for all map-based CP definitions were 0.019-0.027 for Aberdeenshire and 0.014-0.021 for Ayrshire. For CPs defined by <1 km point distance, density was 0.012 for both samples. This was only slightly less than for CPs in Ayrshire defined by a shared boundary excluding those with rivers and ditches between. For Aberdeenshire however, this was about half the density of most of the map-based CP definitions. For CPs defined by <3 km point distance, density was quadrupled in Aberdeenshire when compared to <15 separation of field boundaries, and quintupled in Ayrshire (Table 5). Area-weighted tessellation overestimated density less than <3 km point distance did for both sample networks.

## Discussion

The point locations of farm premises were not completely accurate: distances between the CPH-matched point and field locations were ≥1 km in 8.4% and 8.2% of the sample in Aberdeenshire and Ayrshire, respectively. Overall, <3 km point distance had the most balanced identification of map-based CPs and map-based non-CPs when compared to each the <1 km and <5 km categories, and therefore had the highest TSS score of point distances.

Point distance measures do not seek to classify premises within any given distance as contiguous, rather that they are given a weighted level of risk based on the distance from an IP. By comparing these measures against map-based contiguity as if they also defined contiguity does, however, enable us to begin to consider how accounting for map-based contiguity might alter the shape of the transmission kernel. In reality, during the FMD 2001 outbreak, pre-emptive culling was in part determined by identification of CPs on the ground, since they were considered to be at increased risk of becoming infected. Therefore, if contiguous spread does account for a considerable proportion of transmission events IPs would have an elevated rate of transmission relative to true CPs, regardless of Euclidean point distance between the premises. This would leave transmission events attributable to routes other than those linked to contiguity (e.g. fence line contact, fomites blown between premises), to be captured by the kernel. Crudely, this might be thought of as considering only the relative rate of transmission to map-based non-CPs based on distance between the premises, although in reality map-based CPs would be at risk from these alternative transmission routes as well. Nonetheless this would likely change the shape of the kernel more at small distances than those further away, since at <1 km point distance, an average of 44.9% and 51.9% were map-based non-CPs in Aberdeenshire and Ayrshire, respectively, but at <5 km these figures were 91.4% and 93.9%, respectively. Indeed, once contiguous transmission is separated out from the kernel, it might be the case that another distance measure such as road distance, as previously considered by Savill *et al*. [9], better represents the distance-risk relationship for non-contiguous mechanisms of spread.

In both sample areas, Voronoi tessellation had a slightly lower TSS than for <3 km point distance. Area-weighted tessellation on the other hand had a slightly higher TSS than for <3 km point distance in Aberdeenshire, but slightly lower TSS in Ayrshire. This suggests that, in terms of discrimination between map-based CPs and non-CPs, <3 km point distance and area-weighted tessellation perform similarly, and that the best option may be determined by the landscape of the area that the method is to be applied to. Voronoi and area-weighted tessellation measures performed better overall in Aberdeenshire than in Ayrshire, with somewhat higher TSS scores like-for-like. This may be attributed to sensitivity being considerably poorer in Ayrshire, such that more map-based CPs were being missed by the tessellations. This in turn was likely to be due to the greater density of farm premises in the sample, leading to a greater distortion of contiguity when tessellating around more tightly packed points. Thus in areas of high livestock farm density, tessellation methods may capture contiguity between farm premises with less accuracy than in lower density areas. While the low levels of accuracy (≈20-25%) reported for predicting culled farms by an adapted version of the Keeling *et al.* (2001) model [4] are likely due largely to the complex ‘on the ground’ implementation of culling during the 2001 FMD outbreak, the less than perfect performance of area-weighted tessellation in discriminating between map-based CPs and non-CPs may also have been a contributing factor.

The distances used for field edge based measures in this paper have been used to analyse the persistence of bovine tuberculosis (bTB) [11]. These definitions were far superior to either point distance or tessellation approximations in identifying map-based CPs in the two samples, reflected in their consistently high TSS scores (≥0.868). By definition they captured all of the map-based CPs as these were also calculated based on field edge distance, only using smaller distances of separation. However, PPV indicated that landscape features do interrupt map-based CP boundaries – accounting for up to 29.4% decrease in PPV when all landscape features were taken into account (for Aberdeenshire, from 93.9% for all separated <15 m at field edges to 66.3% for all separated <15 m at field edges excluding those separated by rivers, roads/tracks, and ditches). While this may vary depending on the area of study and the landscape features considered to have an effect on a particular disease’s transmission, it suggests that the way in which premises are perceived to be connected may be substantially altered after taking them into account. Indeed, the mechanism of spread of different diseases must be considered when studying the effects of contiguity. For example, the spread of bTB via badger-to-cattle as well as cattle-to-cattle routes means that extended distances between field edges are likely to be appropriate since badgers can roam freely. However, there is some evidence to suggest that bTB prevalence increases following repeated badger culling are less marked when topographical features such as rivers and motorways are present [19], as these features act as barriers to isolate badger populations. Such features may therefore be worth incorporating into analyses of bTB in cattle populations since they are likely to have a knock-on effect.

Mean degree (i.e. mean number of CPs) and density of map-based CP measures were considerably altered by modifying classification of such CPs by presence of landscape features. When scaled up to a network at the regional or national scale, landscape features could affect contact patterns considerably and therefore potentially also affect transmission of disease through livestock populations. Point distance <1 km created network properties closest to that of map-based CPs, followed by area-weighted tessellation, and then by <3 km point distance. Of note, area-weighted tessellation produced mean degree results in the sample areas (Ayrshire = 6.25; Aberdeenshire = 5.95) similar to that observed over the whole of GB by Keeling *et al.*[3] (6.5, in supplementary information). Therefore, on balance, area-weighted tessellation appears to be better than <3 km point distance at capturing map-based contiguity: it has similar ability to discriminate between map-based CPs and non-CPs and better ability to estimate network density and mean degree. However, one limitation of area-weighted tessellation is that it does not identify the variations in mean degree under map-based CP definitions by livestock species kept on holding (and potentially other predictors of degree as well). This is likely to be important given the differences observed in FMD transmissibility between sheep and cattle during the 2001 outbreak [3].

Notably, the two sample areas showed that the different CP measures performed fairly consistently between them. The Ayrshire sample had a much higher number of farm premises than the Aberdeenshire sample however, and this brought to light some differences in the landscapes. Ayrshire had a higher mean degree than Aberdeenshire for map-based CP definitions, indicating that the livestock farming landscape is less fragmented, and that farm premises on average have more connections to other farm premises. This reflects what is already known about the different farming landscapes of the two areas – Aberdeenshire’s being largely composed of mixed cropping and livestock, and Ayrshire’s being predominantly dairy cattle farming [12]. However, network density is lower in the Ayrshire sample. This is because it has about 72% more farm premises compared to the Aberdeenshire sample, meaning that the total number of possible connections is increased disproportionately to the actual number of connections that exist. The proportion of map-based CPs identified was slightly higher in Ayrshire with <1 km point distance, and slightly lower with <3 km point distance, than compared to Aberdeenshire, both of which may also be attributable to the farming landscape being less fragmented and more tightly-packed with premises in Ayrshire.

In Scotland, Cattle Tracing System (CTS) Links enable premises to move certain livestock freely between paired premises, as described in Orton *et al*. [20]. Given that, in the largest CTS Link that they identified, the majority of premises were in Scotland [20], knowing which premises are linked to one another will likely considerably affect the contact structures of the networks, when scaling up to the national level. Including this information in future analyses would be greatly beneficial.

This paper has considered field contiguity throughout the analysis. However, ultimately it is livestock-inhabited field contiguity that would be the key measure of interest when looking to incorporate contiguity information into analysis of livestock disease spread between premises. The next step will be to create a reliable automated process so that the process of examining contiguity can be extended to larger areas.

## Conclusions

This paper has demonstrated that none of the Euclidean point distance, Voronoi tessellation, or area-weighted tessellation measures discriminate particularly well between map-based CPs and non-CPs as identified from premises field boundaries. Therefore, including contiguity as based on field edges rather than on area-weighted tessellation around farm premises point locations may improve model accuracy. Furthermore, taking topographic features into account can have a considerable impact on which premises are considered to be contiguous or non-contiguous, and on the resulting mean degree and network density. Thus, if such features are known to prevent transmission between contiguous premises (as has been demonstrated for rivers and railways for FMD [10]), including this level of detail could likely also improve the individual farm-level accuracy of model predictions.

## Abbreviations

bTB: Bovine tuberculosis; CP: Contiguous premises; CTS: Cattle tracing system; DC: Dangerous contact; FMD: Foot-and-mouth disease; IP: Infected premises; PPV: Positive predictive value; Se: Sensitivity; TSS: True skill statistic.

## Competing interests

The authors declare that they have no competing interests.

## Authors’ contributions

JSF contributed to the research design, gathered the map-based contiguity data, conducted the analyses, and drafted the manuscript. TP contributed to the research design, and helped to draft the manuscript. MJT conducted the necessary work to identify contiguous premises on the basis of area-weighted tessellation and helped to draft the manuscript. MEJW conceived the research and research design, and helped to draft the manuscript. All authors have read and approved the final manuscript.

## Supplementary Material

Additional file 1 Distribution of distances between point location and nearest field location by point location source. Click here for file

Additional file 2 Concordance (%) of different definitions of being contiguous for sample areas in Aberdeenshire and Ayrshire. Click here for file
