# Supplementary material for: The performance of approximations of farm contiguity compared to contiguity defined using detailed geographical information in two sample areas in Scotland: implications for foot-and-mouth disease modelling
Source: BMC Vet Res. 2013 Oct 8;9:198. doi: 10.1186/1746-6148-9-198 (PMC4126065; doi:10.1186/1746-6148-9-198)
Supplement: Supplementary file 1 [file 1746-6148-9-198-S1.docx]

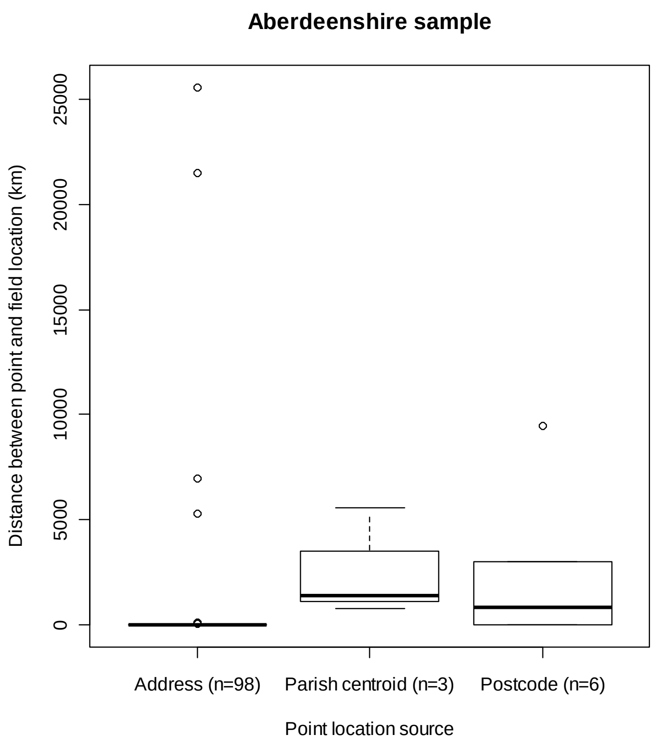
Aberdeenshire sample
Distance between point and field location (km)
0
5000
10000
15000
20000
25000
Address (n=98)
Parish centroid (n=3)
Postcode (n=6)
Point location source

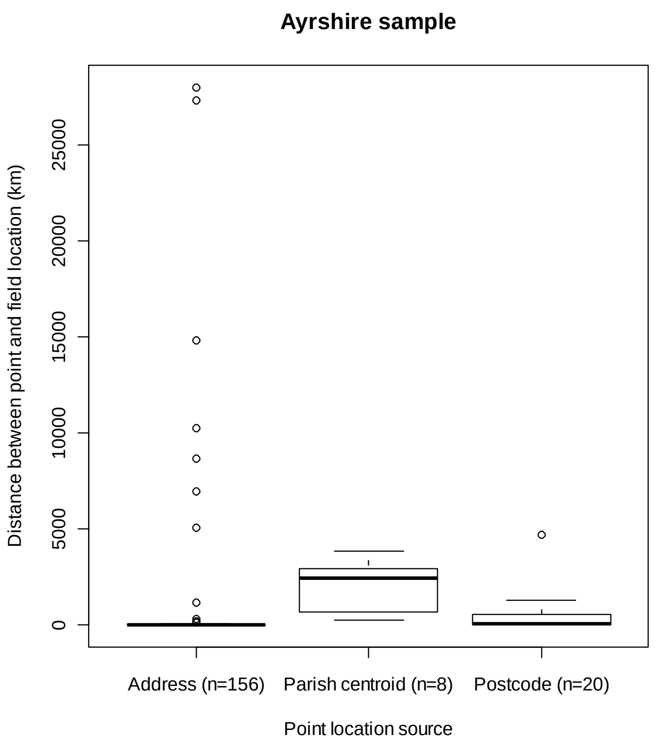
Ayrshire sample
Distance between point and field location (km)
0
5000
10000
15000
20000
25000
Address (n=156)
Parish centroid (n=8)
Postcode (n=20)
Point location source
